# Cognition and connectomes in nondementia idiopathic Parkinson’s disease

**DOI:** 10.1162/NETN_a_00027

**Published:** 2018-03-01

**Authors:** Luis M. Colon-Perez, Jared J. Tanner, Michelle Couret, Shelby Goicochea, Thomas H. Mareci, Catherine C. Price

**Affiliations:** Department of Psychiatry, University of Florida, Gainesville, FL, USA; Department of Clinical and Health Psychology, University of Florida, Gainesville, FL, USA; Department of Medicine, Columbia University, New York, NY, USA; Department of Medicine, University of Florida, Gainesville, FL, USA; Department of Biochemistry and Molecular Biology, University of Florida, Gainesville, FL, USA

**Keywords:** Connectome, Connectivity, Tractography, Structural networks, Parkinson’s disease, Cognitive decline

## Abstract

In this study, we investigate the organization of the structural connectome in cognitively well participants with Parkinson’s disease (PD-Well; *n* = 31) and a subgroup of participants with Parkinson’s disease who have amnestic disturbances (PD-MI; *n* = 9). We explore correlations between connectome topology and vulnerable cognitive domains in Parkinson’s disease relative to non-Parkinson’s disease peers (control, *n* = 40). Diffusion-weighted MRI data and deterministic tractography were used to generate connectomes. Connectome topological indices under study included weighted indices of node strength, path length, clustering coefficient, and small-worldness. Relative to controls, node strength was reduced 4.99% for PD-Well (*p* = 0.041) and 13.2% for PD-MI (*p* = 0.004). We found bilateral differences in the node strength between PD-MI and controls for inferior parietal, caudal middle frontal, posterior cingulate, precentral, and rostral middle frontal. Correlations between connectome and cognitive domains of interest showed that topological indices of global connectivity negatively associated with working memory and displayed more and larger negative correlations with neuropsychological indices of memory in PD-MI than in PD-Well and controls. These findings suggest that indices of network connectivity are reduced in PD-MI relative to PD-Well and control participants.

## Author Summary

Parkinson’s disease (PD) patients with amnestic mild cognitive impairment (e.g., primary processing-speed impairments or primary memory impairments) are at greater risk of developing dementia. Recent evidence suggests that patients with PD and mild cognitive impairment present an altered connectome connectivity. In this work, we further explore the structural connectome of PD patients to provide clues to identify possible sensitive markers of disease progression, and cognitive impairment, in susceptible PD patients. We employed a weighted network framework that yields more stable topological results than the binary network framework and is robust despite graph density differences, hence it does not require thresholding to analyze the connectomes. As Supplementary Information (Colon-Perez et al., 2017), we include databases sharing the results of the network data.

## INTRODUCTION

Parkinson’s disease (PD) is a neurodegenerative disorder characterized by motor disruption (i.e., tremors, unstable posture, bradykinesia) and cognitive symptoms (Chaudhuri & Schapira, 2009). Although James Parkinson initially proposed that PD did not progress to the cerebrum (Parkinson, 2002), there is now substantial evidence that PD can compromise higher cortical cognitive processes. The progression may not be the same for all individuals with PD, however; some individuals remain with primary processing-speed impairments while others may present with primary memory impairments and are at greater risk for dementia (Henderson et al., 2016; Janvin, Larsen, Aarsland, & Hugdahl, 2006). Neuroimaging investigations show regional fractional anisotropy (FA) and volumetric gray and white matter (WM) differences in PD and non-PD peers (Price et al., 2016; Tanner et al., 2015). PD may be a network-level disease (Bellucci et al., 2016; Catani & Ffytche, 2005; Gratwicke, Jahanshahi, & Foltynie, 2015), and the network heterogeneity possibly explains whether the subjects have memory impairment.

Novel applications with structural connectomes hold promise for improving our understanding of network heterogeneity within PD. Connectome studies represent the brain as a set of nodes (brain areas) and edges (connecting white matter between brain areas) that quantify the macroscopic topological organization of the brain network. The topological features of the human connectome allow us to describe the complex interconnectedness of the human brain in vivo. Connectome studies quantitatively describe the arrangement of connections in the brain (Sporns, 2011b) and offer a novel approach to explore the brain in healthy and neuropathological participants (Hagmann et al., 2010; Sporns, 2011a). They have been used to quantify the organization of connected white matter in neurological disorders such as Huntington’s disease (Odish et al., 2015), epilepsy (Taylor, Han, Schoene-Bake, Weber, & Kaiser, 2015), and Alzheimer’s disease (Daianu et al., 2015). Also, connectome topology has been suggested as a sensitive biomarker for early stages of psychotic illness and the eventual development of psychosis (Drakesmith et al., 2015). In individuals with PD who have a freezing gait, maladaptive brain restructuring has been shown through the connectivity between locomotor hubs, particularly in the supplementary motor area and mesencephalic locomotor regions (Fling et al., 2014). A recent study showed that the connectome in patients with PD with mild cognitive impairment (MCI) is altered (Galantucci et al., 2016). Hence, further structural connectome studies of PD may yield sensitive markers of disease progression, cognitive impairment, and susceptibility to PD.

In this work, we performed global (average values for the entire brain network for each participant) and local (average values for every individual node for each participant) connectome analyses. Given the elevated risk of developing dementia associated with amnestic mild cognitive impairment in PD (Henderson et al., 2016), in this study we examined structural connectome differences in people with PD who are cognitively well relative to individuals with PD who meet criteria for amnestic MCI. We also explored the correlations between topological connectome indices and the most common cognitive vulnerabilities of PD (i.e., processing speed, working memory, and episodic memory; Zgaljardic, Borod, Foldi, & Mattis, 2003). We used diffusion MRI (Basser & Jones, 2002) and tractography (Basser, Pajevic, Pierpaoli, Duda, & Aldroubi, 2000) to generate connectomes comprising 82 brain regions (68 cortical and 14 subcortical). With this, we determined the organization of structural connectivity of our three participant groups (PD, PD-MCI, non-PD peers) and correlations between connectome indices and working memory, processing speed, and verbal memory.

## METHODS

### Participants

This study was approved by the University of Florida Health Center Institutional Review Board (Protocol #472-2007). Written consent was obtained from all participants, and all research followed the Declaration of Helsinki.

Providers within the UF Center for Movement Disorders and Neurorestoration referred nondemented individuals with idiopathic PD to the study. Structured telephone screening was performed to verify inclusion/exclusion criteria. Potential participants were screened in person with the Dementia Rating Scale-2 (DRS-2) (Matteau et al., 2011); a total DRS-2 score > 130 was required for participation. Only nondemented individuals who were able to consent to participate were included in the study. Inclusion criteria: right-handed (Briggs & Nebes, 1975), DRS-2 raw score > 130, fluent English, diagnosis of PD by a movement disorder neurologist, UK Parkinson’s Disease Society Brain Bank Clinical Diagnostic Criteria (Hughes, Ben-Shlomo, Daniel, & Lees, 2001), and Hoehn and Yahr scale (Hoehn & Yahr, 1967) ranging from 1 to 3. Exclusion criteria: diseases likely to confound cognition (e.g., cerebrovascular accident in the last six months), deep brain stimulation, secondary/atypical Parkinsonism, and major psychiatric disorder. Depression and apathy were not exclusion criteria because of their high prevalence in PD.

The final sample included 40 people with idiopathic PD and 40 non-PD peers. Diffusion and gray matter structural data from some of these participants have also been seen in recent publications (Crowley et al., 2017; Price et al., 2016; Schwab et al., 2015; Tanner, Levy, et al., 2017; Tanner et al., 2015; Tanner, McFarland, et al., 2017).

### Defining PD Subgroups

PD-Memory Impaired (PD-MI): From the measures described below, those with PD who had a memory composite score ≤ −1.5 (relative to non-PD peers) were classified as PD-MI (*n* = 9). All others with PD were classified as PD without memory impairment (PD-Well; *n* = 31). Those with and without PD-MI are discussed elsewhere (Tanner et al., 2015).

### Cognitive Measures

While on medication, participants completed cognitive testing, neuroimaging, and the Unified Parkinson’s Disease Rating Scale (UPDRS) to assess optimal performance and represent normal functioning. All participants also completed tests on general cognition and mood, PD symptoms and severity, comorbidity (Charlson, Pompei, Ales, & MacKenzie, 1987), and a neuropsychological protocol. Medications were reverted to a common metric (levodopa equivalency dose, LED; Tomlinson et al., 2010). Blinded raters scored the data twice. The UPDRS III is a measure of PD motor symptom severity and was used as a correlate with network indices.

Primary cognitive domains of interest: PD is brain disorder of the frontal-subcortical areas known to alter the frontal-striatal cognitive functions of processing speed and working memory. These domains were assessed using composites of standardized neuropsychological measures:

•  Processing speed: based on standardized scores from the Trail Making Test, Part A (total time; Heaton & Psychological Assessment Resources Inc., 2004), WAIS-III Digit Symbol (total correct; Wechsler, 1991), and Stroop Color Word Test–Word Reading condition (total correct; Golden & Freshwater, 2002).
•  Working memory: created from the Digit Span Backward (total span; Wechsler, 1991), Spatial Span Backward (total score; Wechsler, 1991), and Letter Number Sequencing (total correct; Wechsler, 1991).

In addition to frontal-striatal deficits, we examined connectome indices relative to declarative memory abilities, as this is an essential domain of prodromal dementia.

•  Verbal memory: created from selected index scores from the 12-word version of the Philadelphia (repeatable) Verbal Learning Test (P(r)VLT (Price et al., 2009) and Wechsler Memory Scale 3rd Revision (WMS-III) Logical Memory (LM) (Wechsler, 1991).

### MRI Acquisition and Processing

Data were acquired with a Siemens 3 T Verio using an eight-channel head coil. Two T1-weighted scans were used for node segmentation with scan parameters of 176 contiguous slices, 1 mm^3^ isotropic voxels, and TR/TE = 2,500/3.77 ms. Single-shot echo planar imaging diffusion-weighted images were acquired for tractography with gradients applied along 6 (*b* = 100 s/mm^2^) and 64 directions (*b* = 1,000 s/mm^2^). Diffusion imaging parameters were set at 73 contiguous axial slices with 8 mm^3^ isotropic voxels and TR/TE = 17,300/81 ms. Node segmentation was completed with FreeSurfer 5.3 and data were quality checked. The quality check for the diffusion data included visual inspection for artifacts (e.g., signal loss in the anterior and middle regions, Venetian blinding, checker boarding). No significant artifacts were observed. The process was also repeated after eddy current correction (eddy_correct). Participant head motion during diffusion sequences was quantified with four measures using TRACULA (Yendiki, Koldewyn, Kakunoori, Kanwisher, & Fischl, 2013). Between-group registration and intensity-based metrics demonstrated no significant group differences in diffusion sequence motion (Registration: average translation: *t* = 0.98, *p* = 0.33; average rotation: *χ*^2^ = 1.25, *p* = 0.26; Intensity: Percentage bad slices *χ*^2^ = 0.26, *p* = 0.61; Average dropout score *χ*^2^ = 0.26, *p* = 0.61), suggesting that data were appropriate for group comparisons.

Diffusion data were preprocessed using in-house software written in IDL (Harris Geospatial Solutions, Bloomfield, CO). Eddy current correction was performed using FSL (Jenkinson, Beckmann, Behrens, Woolrich, & Smith, 2012). Diffusion tensor imaging metrics (fractional anisotropy, FA, and mean diffusivity, MD) were calculated using FSL. For tractography, fiber orientation profiles were estimated based on the calculation of diffusion displacement probability with a mixture of the Wishart method outlined by Jian, Vemuri, Ozarslan, Carney, and Mareci (2007). Diffusion images were interpolated (Meijering, Zuiderveld, & Viergever, 1999) to 1 mm^3^ isotropic voxels using cubic convolution and whole-brain deterministic fiber tracking initiated using 125 uniformly distributed streamline points per voxel.

### Network Preparation and Analysis

The network edges were weighted as described by Colon-Perez et al. (Colon-Perez, Spindler, et al., 2015). The edge weight, *w*(*e*), defines connecting any two nodes is defined as

$$w(e_{ij})=\left(\frac{V_{voxel}}{P_{voxel}}\right)\left(\frac{2}{A_{i}+A_{j}}\right)\overset{P_{voxel}}{\underset{p=1}{\sum }}\overset{M}{\underset{m=1}{\sum }}\frac{\chi_{R}(f_{p,m})}{l(f_{p,m})},$$ (1)

where

$$\chi_{R}(f_{p,m})=\left\{\begin{array}{l} 1,f_{p,m}\in R\\ 0,f_{p,m}\notin R \end{array}\right.\;,$$ (2)

*V*_*voxel*_ is the MR voxel volume, *P*_*voxel*_ is the number of streamline seed points per voxel, *A* is the surface area of each node, *M* is the number of voxels making up the edge, *f*_*p*,*m*_ is the streamline originating from seed point *p* in voxel *m*, *l*(*f*_*p*,*m*_) is the length of *f*_*p*,*m*_, *R* is the set of streamlines that originate from the voxels making up the space occupied by the WM path connecting nodes *n*_*i*_ and *n*_*j*_, and *χ*_*R*_(*f*_*p*,*m*_) is the characteristic function that ensures the streamlines connecting nodes *n*_*i*_ and *n*_*j*_ originate from the space (i.e., voxels) the streamlines traverse between nodes *n*_*i*_ and *n*_*j*_. The edge weight (Equation 1) eliminates the bias effects of the length of the streamlines, the seeding paradigm (i.e., *P*_*voxel*_), image resolution (i.e., *V*_*voxel*_), and tractography-specific experimental factors from the calculation; for more details we refer the reader to Colon-Perez, Spindler, et al. (2015). The characteristic function (*χ*_*R*_(*f*_*p*,*m*_), Equation 2) eliminates those streamlines that originate within the nodes and voxels that do not represent the WM path connecting the nodes. Also, this edge weight quantifies the white matter strength between any two nodes in a dimensionless and scale-invariant manner (Colon-Perez, Spindler, et al., 2015). The employed edge weight uses the strict criterion of *χ*_*R*_(*f*_*p*,*m*_) to determine the set streamlines used to quantify the strength of connectivity between two nodes. Given the high level of false positives in tractography, this edge weight serves as a layer of strict control to quantify the strength of connectivity between nodes.

The networks were analyzed in a weighted framework, as described in Colon-Perez, Couret, Triplett, Price, and Mareci (2016). This approach is similar to the binary framework used by Watts and Strogatz (1998), but with an additional degree of freedom from the edge weighting (Equation 1). In our previous work, we showed that this weighted framework yields topologically relevant features without the need for thresholds, therefore no threshold was applied to generate the brain connectomes in this work (Colon-Perez et al., 2016). Weak edges in a network are thought to provide a cohesive strength to networks (Granovetter, 1973). The ability to obtain stable connectome results across thresholds, shown in Colon-Perez et al. (2016), allows us to maintain these weak edges in our analysis. These networks were studied with the following indices: (a) *graph density* (Boccaletti, Latora, Moreno, Chavez, & Hwang, 2006), which is a binary metric that quantifies the fraction of edges in a graph (only *nonweighted* index in this study); (b) *node strength* (Newman, 2001), which is a weighted topological index of the relative connectivity strength of the nodes with the rest of the network; (c) *clustering coefficient* (Zhang & Horvath, 2005), which is a weighted metric that quantifies the strength of connectivity between the neighbors of a node; (d) *path length* (Colon-Perez et al., 2016), which is a weighted metric that quantifies the strength of the shortest path between two nodes; and (d) *small-worldness* (Humphries & Gurney, 2008), which is a weighted metric that estimates the likelihood that networks display similar path lengths and higher clustering to a network connected by randomly assigned edges, as described by Erdös and Rényi (1959). For a complete description of the weighted network analysis, refer to Colon-Perez et al. (2016). The results of these network indices in this manuscript will be referred to as global for each participant when the results are averaged into a single value for the entire brain network (yields one value per participant). The local results for each participant refer to the average values per node (yields 82 values per participant). Previous studies described global differences in node strength and path length in patients with PD (Galantucci et al., 2016); in this work, we use these indices to identify local changes in network connectivity in addition to corroborate previous global changes described by Galantucci et al. (2016). The clustering coefficient is reduced globally in patients with PD (Luo et al., 2015); in this study, we identify the local changes in clustering coefficient and identify those nodes responsible for the reduction. Several PD studies have confirmed the small-world topology in controls and patients with PD; in this study, we assess whether PD-Well or PD-MI displays a difference from controls.

### Statistics and Correlations

Cognitive composites and network variables were tested for statistical significance with a nonparametric Mann-Whitney test using R (version 3.1.3) (R Core Team, 2015). All node-specific network results were controlled by the graph density to control the influence of global connectivity features in local indices (i.e., node-specific; van Wijk, Stam, & Daffertshofer, 2010). To correct for multiple comparisons, we used the “fdrtool” package (Strimmer, 2008) and the false nondiscovery rate, which estimates the proportion of nondiscovery rejections or type II errors (Genovese & Wasserman, 2002). This tool works by first finding a suitable cutoff point using an approximate null model, which is fitted; subsequently, a cutoff point is sought with a false nondiscovery rate as small as possible. Scale parameters of the null model and proportion of null values are then estimated from the data. The corresponding *p* values are computed, and a modified Grenander algorithm (The Grenander, 1956, R implementation in fdrtool can be found in https://cran.r-project.org/web/packages/fdrtool/fdrtool.pdf) is used to find the overall density and distribution function. Finally, adjusted *p* values are determined and reported. Correlations were calculated between network indices (e.g., node strength, path length, clustering, small-worldness) and neuropsychological composites (e.g., working memory, memory speed, and verbal memory). Since our goal is to identify the network indices that correlate with the different neurocognitive domains under study, no correlations were assessed between the various network indices. The correlations were estimated using a Spearman’s partial correlation method controlling for education level using the “ppcor” package in R. With significance in our correlative analysis set at *α* = 0.05 and given our sample size of *N* = 80, we decided to reduce potential false positives by considering only correlations larger than 0.50 (Cohen, 1992).

## RESULTS

### Demographics

Control and PD groups were not statistically different in their average age (Table 1). The PD-Well group did not show a significant difference from controls in education, working memory, or memory composites (Table 1).

**Table 1.** Demographics and cognitive indices

	**Control (*n* = 40)**		**PD-Well (*n* = 31)**		**p**	**PD-MI (*n* = 9)**		**p**
	**Mean**	***σ***	**Mean**	***σ***		**Mean**	***σ***	
Age (yrs)	68.18	4.64	67.3	5.02	0.38	69.4	6.77	0.60
Duration (yrs)	NA	NA	7.87	5.60	NA	6.22	3.07	NA
Education (yrs)	16.75	2.35	16.8	2.91	0.86	14.3	2.74	8.23E-3*
UPDRS III	2.75	3.36	18.2	11.6	1.34E-10*	15.7	7.25	4.52E-6*
Working mem	1.15	0.55	0.89	0.77	0.148	0.27	0.57	6.70E-5*
Proc speed	0.16	0.47	−0.42	0.56	3.23E-5*	−0.68	0.76	3.90E-3*
Mem comp	0.00	1.00	−0.32	0.70	0.082	−1.76	0.20	3.54E-5*

Statistical significance was tested between PD subgroups (i.e., PD-Well and PD-MI) and controls. Yrs = years, Duration = disease duration, UPDRS = Unified Parkinson’s Disease Rating Scale, Working mem = working-memory score, Proc speed = processing-speed composite score, Mem comp = memory composite score, and *σ* = standard deviation. Statistics performed with Mann-Whitney statistics between PDs and controls. * correspond to statistically significant differences.

The PD-Well participants’ working-memory scores did not show significant deficits, whereas PD-MI was 0.88 standard deviations lower in working-memory score. The PD-MI group was 2.5 years less educated than controls (Table 1) and PD-Well (*p* = 0.03, *p* value obtained between PD-MI and PW-Well). Relative to controls, PD-Well and PD-MI displayed a reduction in their processing-speed scores (Table 1).

The combined PD group (*n* = 40) had lower memory composite scores than controls (*p* < 0.01). PD-Well had lower memory composite scores than controls, but this was not statistically significant (*p* = 0.082; Table 1). PD-MI had lower memory composite scores than both cohorts: controls (*p* < 0.001) and PD-Well (*p* < 0.001; Table 1). Group differences also remained after controlling for processing speed and working memory (*p* < 0.001).

### Global Network Measures

#### Results and differences

**Figure 1.**
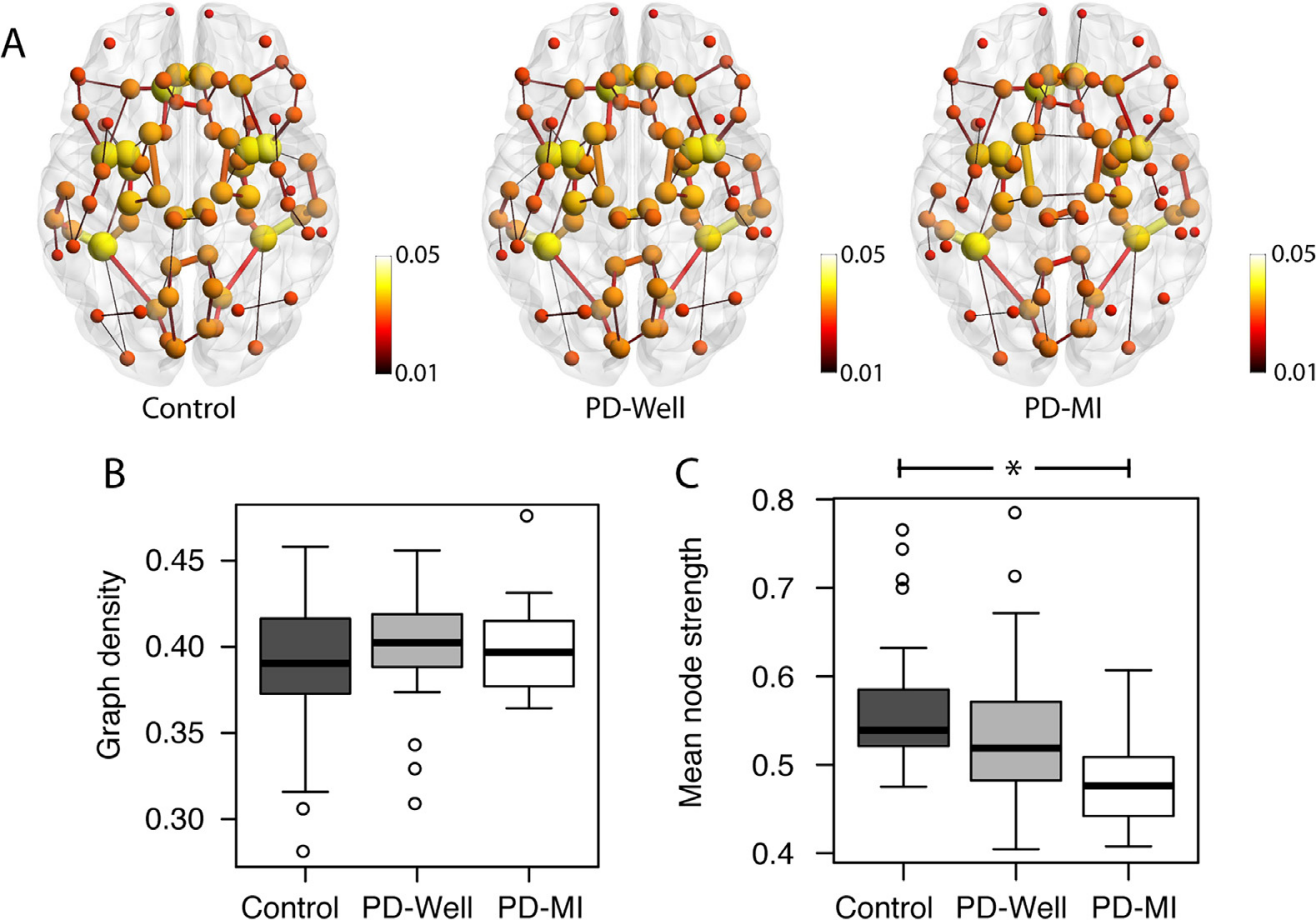
The structural connectivity of controls, PD-Well, and PD-MI participants. (A) Average connectome per group; the node size represents the strength of the connections with the rest of the network, and the edge width represents the relative strength of connections between pairs of nodes. (B) Box-plot distributions of graph density values (number of connections in connectome). No significant differences in the number of edges were observed between groups. (C) Box-plot distributions of average global node strength. The mean node strength group comparison between Control and PD-MI was significantly reduced (*p* = 0.002). The connectome images in this figure were prepared using BrainNet (Xia, Wang, & He, 2013).

**Figure 2.**
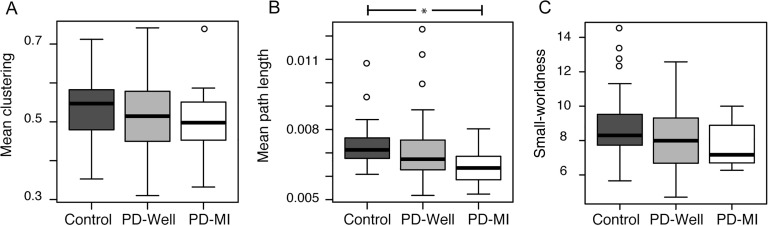
Box plots of global network indices for all groups. (A) Mean clustering = mean global clustering coefficient per brain averaged across subjects within each group. (B) Mean path length = mean global path length per brain averaged across subjects within each group. (C) Small-worldness = small-worldness index per brain averaged across subjects within each group. * = statistically significant difference (*p* < 0.05), ° = outliers. Statistics performed with a nonparametric Mann-Whitney test.

The connectomes in Figure 1A show the top 1% of the strongest connections and the connectivity alterations in the PD-MI connectomes relative to controls. The node size and color represent the node strength, and the edge thickness represents the edge weight value. In particular, connections between nodes in the temporal lobe are smaller in size (representing a reduction in edge weight). The graph density (Figure 1B) in all groups was approximately 40% of all possible edges, with no significant differences between controls and PD-Well (*p* = 0.249) or between controls and PD-MI (*p* = 0.624). To reduce the bias of graph density on network indices, these will be controlled by the graph density for the rest of the manuscript (van Wijk et al., 2010). A 4.99% reduction in mean node strength is observed over the entire network between controls and PD-Well (*p* = 0.041), whereas mean node strength in the PD-MI group was reduced relative to controls by 13.23% (*p* = 0.004; Figure 1C). The mean path lengths for the entire network were reduced by 1.97% between controls and PD-Well (*p* = 0.058), whereas the mean path lengths in the PD-MI group displayed a significant decrease of 11.7% (*p* = 0.014, Figure 2B). The mean clustering coefficient for the entire network did not differ significantly between controls and PD-Well (*p* = 0.162) or PD-MI (*p* = 0.320), with mean values approximately 0.55 (Figure 2A). The small-worldness index did not differ significantly between controls and PD-Well (*p* = 0.188) or PD-MI (*p* = 0.131), with mean values approximately 8.00 (Figure 2C). Also, we determined node strength, clustering coefficient, and path length with a more traditional weighting scheme of FA as the edge weight. We did not find any differences between controls and PD-Well or controls and PD-MI using FA as the edge weight (all *p* > 0.1; the database is available as an online supplement and Supplementary Figure 3; see Colon-Perez et al., 2017).

#### Correlations

For the controls, global network indices did not show any correlations with the primary neuropsychological composites of interest (Table 2). The memory composite score did not correlate with any network index for any of the groups. The working-memory composite score showed negative correlations with node strength for PD-Well participants and control. Path length also showed a negative correlation with working memory between PD-Well and controls. The PD-MI group showed negative correlations between all network indices and the processing-speed composite (except small-worldness); working memory negatively correlated with path length. For PD-MI, there were positive correlations between the Unified Parkinson’s Disease Rating Scale (UPDRS) Part III (motor test) and node strength, as well as path length (Table 2; scatter plots are shown in Supplementary Figure 2; see Colon-Perez et al., 2017).

**Table 2.** Correlations (Fisher’s z-score) between global network indices and neuropsychological composites

**Network index**	**Controls (*n* = 40)**			
	**UPDRS**	**Working mem**	**Proc speed**	**Mem comp**
Node strength	NA	0.225	0.021	0.230
Clustering	NA	0.079	0.149	0.195
Path length	NA	0.100	0.051	0.082
Small-worldness	NA	−0.144	−0.076	−0.148
**Network index**	**PD-Well (*n* = 31)**			
	**UPDRS**	**Working mem**	**Proc speed**	**Mem comp**
Node strength	0.008	−0.574*	0.169	−0.179
Clustering	−0.287	0.018	0.251	0.076
Path length	0.070	−0.594*	0.193	−0.229
Small-worldness	−0.172	0.115	0.230	−0.049
**Network index**	**PD-MI (*n* = 9)**			
	**UPDRS**	**Working mem**	**Proc speed**	**Mem comp**
Node strength	0.824#	−0.314	−0.594#	0.116
Clustering	−0.071	−0.328	−0.685*#	0.356
Path length	0.955#	−0.826*	−0.724*#	0.312
Small-worldness	−0.084	−0.109	−0.450	0.079

UPDRS = Unified Parkinson’s Disease Rating Scale, Working mem = working-memory score, Proc speed = processing-speed composite score, Mem comp = memory composite score, and clustering = clustering coefficient score. Partial correlation controlled for age using Spearman’s method transformed to Fisher’s z-scores using the “psych” package in R. Black bins correspond to |z| > 0.55 and correlated results. * corresponds to statistically significant difference to controls; # corresponds to statistically significant difference to PD-Well, calculated from *r* values with http://vassarstats.net/rdiff.html.

### Local Network Measures

#### Group differences

**Figure 3.**
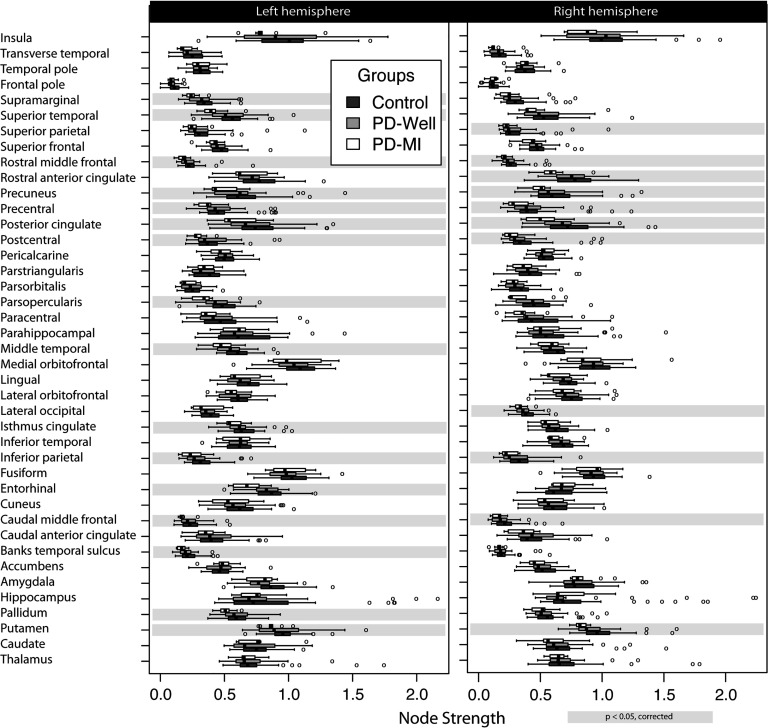
Node strength results in a connectome of 82 cortical and subcortical nodes. Controls (*n* = 40), Parkinson’s participants without memory impairment (*n* = 31), and Parkinson’s participants with memory impairment (*n* = 9). The left column represents the left hemisphere nodes, and the right column corresponds to the right hemisphere. Highlights in gray represent a significant difference (corrected for multiple comparisons) between controls and PD-MI. No significant differences were observed between PD-Well and controls. Banks temporal sulcus is an abbreviation for the banks of the superior temporal sulcus region, which is the name in the FreeSurfer nomenclature; the full name is used in the main text.

**Figure 4.**
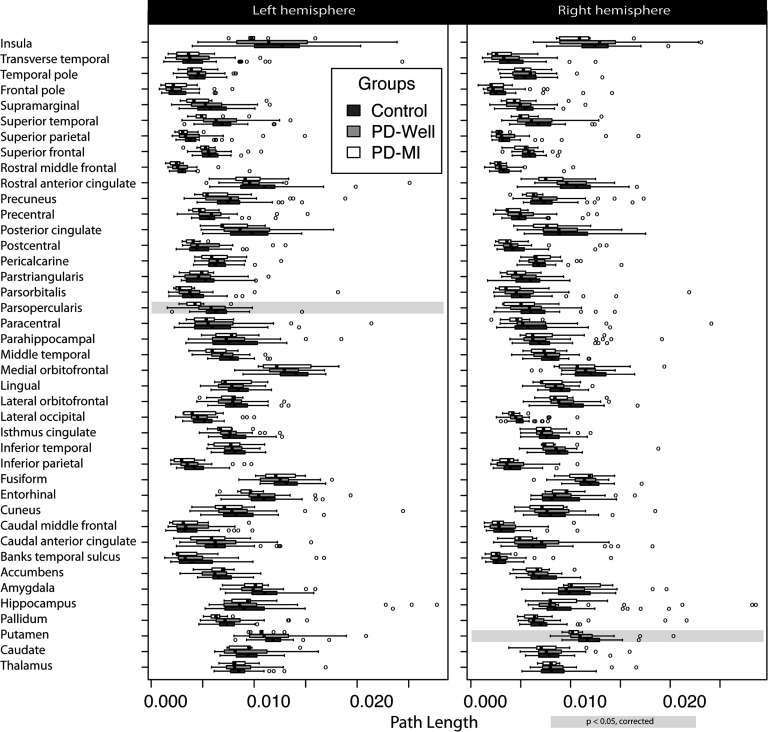
Path length results per node in a connectome of 82 cortical and subcortical nodes. Controls (*n* = 40), Parkinson’s participants without memory impairment (*n* = 31), and Parkinson’s participants with memory impairment (*n* = 9). The left column represents the left hemisphere nodes, and the right column corresponds to the right hemisphere. Highlights in gray represent a significant difference (corrected for multiple comparisons) between controls and PD-MI. No significant differences were observed between PD-Well and controls. Banks temporal sulcus is an abbreviation for the banks of the superior temporal sulcus region, which is the name in the FreeSurfer nomenclature; the full name is used in the main text.

Node strength and path length box plots for each group are shown in Figures 3 and 4. There were local network (i.e., node-specific) differences between controls and PD-MI (no difference between controls and PD-Well; for specific node results, refer to tables in the Supplementary Information, Colon-Perez et al., 2017). After correcting for multiple comparisons and controlling for graph density, statistically significant changes were observed for 27 distinct nodes in node strength (Figure 3), and two nodes for path length (Figure 4). There were no observed differences in clustering for any node. The left (Lf) pars opercularis and right (Rt) putamen showed statistical differences in node strength and path length. The location of the nodes with statistically significant differences can be seen in Supplementary Figure 1.

Bilateral differences in node strength between PD-MI and controls were found for the following: putamen (*p*_Lf_ = 0.043 and *p*_Rt_ = 0.002), caudal middle frontal (*p*_Lf_ = 0.018 and *p*_Rt_ = 0.040), inferior parietal (*p*_Lf_ = 0.031 and *p*_Rt_ = 0.019), postcentral (*p*_Lf_ = 0.017 and *p*_Rt_ = 0.045), posterior cingulate (*p*_Lf_ = 0.043 and *p*_Rt_ = 0.006), precentral (*p*_Lf_ = 0.017 and *p*_Rt_ = 0.019), and precuneus (*p*_Lf_ = 0.019 and *p*_Rt_ = 0.043). Additional nodes with statistically significant differences were the following: Lf pallidum (*p* = 0.021), Lf entorhinal cortex (*p* = 0.004), Lf isthmus cingulate (*p* = 0.027), Lf middle temporal (*p* = 0.016), Lf pars opercularis (*p* = 0.003), Lf banks of the superior temporal sulcus (*p* = 0.019), Lf supramarginal (*p* = 0.002), Lf rostral middle frontal (*p* = 0.003), Lf superior temporal (*p* = 0.006), Rt lateral occipital (*p* = 0.029), Rt rostral anterior cingulate (*p* = 0.019), Rt rostral middle frontal (*p* = 0.007), and Rt superior parietal (*p* = 0.040). The path length was different for Lf pars opercularis (*p* = 0.002) and Rt putamen (*p* = 0.001). Local indices of node strength, clustering coefficient, and path length did not show any differences between controls and PD-Well or controls and PD-MI using FA as the edge weight after correcting for multiple comparisons.

#### Correlations

Given the large number of correlations (three network indices, three cognitive indices, and UPDRS scores for PDs, 82 nodes, and three subject groups), the discussion is restricted to the correlation analysis of those nodes that showed statistically significant differences between controls and PD-MI. Spearman correlations were performed for 27 nodes (i.e., those with statistically significant differences between PD-MI and controls; Figure 3), three network indices, and three neuropsychological indices (PD participants also include UPDRS scores). This analysis yields a maximum of 243 correlations in controls and 324 for individuals with PD (the correlations for all nodes can be found in the online supplemental data; Colon-Perez et al., 2017).

**Figure 5.**
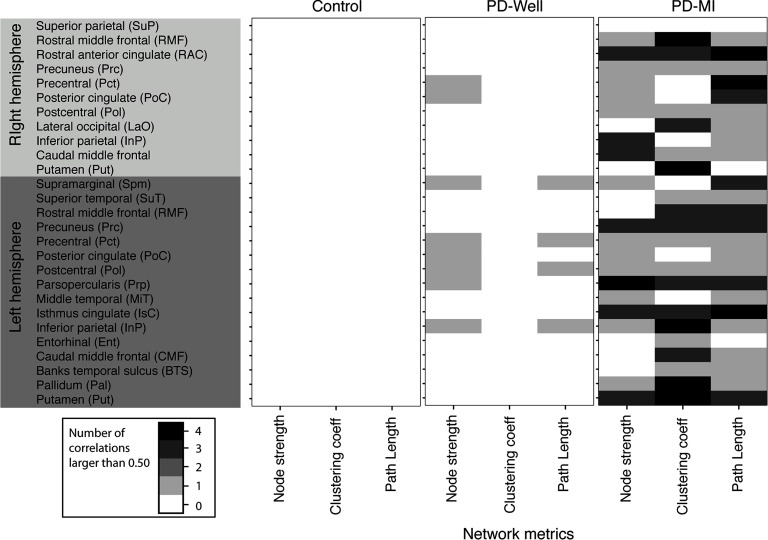
The number of significant correlations (absolute value |r| > 0.5) between network indices and each neuropsychological composite and UPDRS Part III scores (i.e., motor symptoms of PD). The intensity of each pixel is the number of correlations larger than 0.5 for each network metric and the specified node with the neuropsychological composites (i.e., working memory, memory composite, speed composite, and UPDRS Part III). There is a maximum of four possible significant correlations, computed between a single network metric and the four neurocognitive measures. This is then reported for each node with significant differences in node strength between PD-MI and controls (see Figure 3). Banks temporal sulcus is an abbreviation for the banks of the superior temporal sulcus region, which is the name in the FreeSurfer nomenclature; the full name is used in the main text.

Controls: The control group did not exhibit significant correlations between any network metric (i.e., node strength, clustering, or path length) and any neuropsychological composite (working memory, memory composite, or processing-speed composite). See Figure 5, left column.

PD-Well: There were 12 correlations with coefficient values larger than 0.5 between network indices and all neuropsychological composites (composites described in cognitive measures). See Figure 5, middle column. For PD-Well, the node strength correlated with the composites for the following nodes: Lf inferior parietal, Lf pars opercularis, Lf postcentral, Lf posterior cingulate, Lf precentral, Lf supramarginal, Rt posterior cingulate, and Rt precentral. The clustering coefficient did not correlate with any composite. The path length correlated for the Lf inferior parietal, Lf precentral, Lf postcentral, and Lf supramarginal.

PD-MI: There were 63 correlations with values larger than 0.5 between network indices and all neuropsychological composites. The majority of nodes correlated with at least one neuropsychological composite for the PD-MI group (Figure 5); therefore, in this section, we will list the nodes that did not show correlations between network indices and composites. The node strength of PD-MI did not correlate with Lf banks of the superior temporal sulcus, Lf caudal middle frontal, Lf entorhinal, Lf rostral middle frontal, Lf superior temporal, Rt putamen, Rt lateral occipital, and Rt superior parietal. The clustering coefficient did not correlate with composites for Lf middle temporal, Lf posterior cingulate, Lf supramarginal, Rt inferior parietal, Rt precentral, Rt superior parietal, and Rt posterior cingulate. The path length did not correlate for the Lf entorhinal cortex, Rt putamen, and Rt superior parietal.

## DISCUSSION

In this study, we identified differences in the connectome organization for people with idiopathic PD with mild amnestic disturbance (PD-MI), and we explored correlations between connectomes and the most vulnerable cognitive domains within PD relative to non-PD peers. Global differences were identified in the PD-MI versus control groups, in mean node strength and path length but not in clustering coefficients, small-worldness, or graph density. No differences were observed for any of the indices (global or local) for the PD-Well group versus controls. We found that 27 out of 82 nodes showed local differences of connectivity in node strength in the PD-MI group relative to controls (Figure 3). Local node network differences in node strength *and* path length were found in PD-MI brains and were specific to the Lf pars opercularis, and Rt putamen (Supplementary Figure 1). Bilateral differences between controls and PD-MI were found in node strength for the putamen, caudal middle frontal, inferior parietal, postcentral, posterior cingulate, rostral middle frontal, precentral, and precuneus. The right putamen was the only subcortical region that displayed a significant connectome alteration in the form of a reduction in node strength and path length. Node strength and cognitive composites further showed a potential large-scale network connectivity reduction in PD-MI relative to normal cognitive areas of vulnerability. This study shows key connectome indices for consideration in PD-Well and PD-MI phenotypes.

### Structural Differences

Robust connectome differences between PD-MI and controls were observed at the global level with mean node strength and mean path length. In this study, groups’ structural connectomes showed many similar levels of connectivity, as shown in the weighted top edges in Figure 1A and their graph densities in Figure 1B. Relative to non-PD peers, mean node strength was altered by 5% and 13% for PD-Well and PD-MI, respectively, and mean path length was changed 2% and 11%, respectively. These findings in node strength and path length suggest a reduction in the integrity of white matter connectivity in PD. The path length alteration in PD is directly related to the node strength reduction (i.e., *r* = 0.92). The edges (i.e., edge weights) connecting nodes that in turn yield the shortest path between nodes possibly decreases because of the neurodegeneration of white matter in PD (Tessitore, Giordano, Russo, & Tedeschi, 2016). The absence of group differences in clustering coefficient and small-worldness, which is confirmed by a previous report (Galantucci et al., 2016), may suggest a method to compensate for PD changes where the brain network adapts to preserve its small-worldness and clustering features. Therefore, the changes in node strength and path length could be markers of PD progression and cognitive decline. Although cross-sectional and preliminary, these findings suggest a local and global network reduction in connectivity with an amnestic disturbance (i.e., PD-MI).

To further explore the relevance of particular node alterations, we performed local topological analyses that revealed evidence of local network disruption in PD-MI. Node strength and path length abnormalities were seen with the left pars opercularis and right putamen. The frontal region is involved in networks of language, attention, and working memory (Lezak, 2012; Petrides, 2000; Stuss et al., 2005; Zola-Morgan & Squire, 1986; Zola-Morgan, Squire, & Amaral, 1986), while the subcortical nuclei of the putamen is involved in PD motor symptomatology (Braak, Ghebremedhin, Rub, Bratzke, & Del Tredici, 2004; Lisanby et al., 1993; Nemmi, Sabatini, Rascol, & Peran, 2015; Price et al., 2016). Although preliminary, these connectivity reductions primarily in left cortical areas validate the profile of our PD-MI, particularly given that the PD-MI group was classified with verbal memory measures, which involve left hemisphere regions more than the right (Golby et al., 2001). This profile is also consistent with other reports showing anterior and lateral temporal thinning and volume reduction in PD-MI (Crowley et al., 2017; Pagonabarraga et al., 2013; Tanner et al., 2015).

At this point, we would like to illustrate a larger issue in the connectomics literature: the lack of a ground truth edge weight. In this work, we found a discrepancy between our weighted framework (Colon-Perez et al., 2016) and an FA weighting scheme. Several works have been published that bring attention to the inadequacies of traditionally used edge weighting schemes in network neuroscience. Cheng et al. (2012) first described how increasing seed density improves the stability of network metrics. However, there is a caveat that higher seed densities lead to a larger number of spurious streamlines and thus affect the connectome. In our edge weight scheme, we employ a large number of seeds per voxel to increase its stability and use the characteristic function (Equation 2) to mitigate the effects of spurious streamlines (Colon-Perez, Spindler, et al., 2015). Buchanan, Pernet, Gorgolewski, Storkey, and Bastin (2014) suggest that some measure of streamline density (as our edge weight) is superior to FA since it produces better test-retest performance. These biases and others are attempted to be controlled or reduced by our weighting scheme (Colon-Perez, Spindler, et al., 2015). Our work and that of many others are under way to develop new and better ways to weigh networks in connectome studies that improve the stability of network metrics derived from tractography (Colon-Perez et al., 2016; Colon-Perez, Spindler, et al., 2015; Girard, Whittingstall, Deriche, & Descoteaux, 2014). We are not claiming that our weighting scheme is a more accurate representation of the underlying anatomical connectivity than others; but with our previous articles and this one, we hope to continue the discussion to find new and novel ways to weigh connectomes.

Functional MRI studies have shown that disruptions in the motor networks of patients with PD correlate with disease severity (Wu et al., 2009). These functional changes are likely accompanied by structural disruption, potentially like the ones described here. Microstructural changes have been reported in PD using diffusion tensor imaging. Lower FA has been observed in the substantia nigra and the striatum in patients with PD (Tessitore et al., 2016). In contrast, FA increases have been noted in the corpus callosum and the superior longitudinal fasciculus (Gattellaro et al., 2009). This white matter change leads us to hypothesize that interhemispheric connectivity alterations are mediated via the corpus callosum and within-hemisphere alterations through the superior longitudinal fasciculus (Northam et al., 2012). Reductions in FA have also been reported in participants with PD in the putamen, substantia nigra, striatum, frontal lobes, and motor areas (Zhan et al., 2012). These brain connectivity changes are not only restricted to MRI but also have been observed with SPECT (Booij et al., 1997) and PET (Brooks, 1995). Altogether, the research findings, including our study, suggest many changes in PD may coalesce into a reorganization of the structural brain connectome. We recognize that these connectome differences may not be unique to memory-impaired PD because similar differences have been reported in patients with epilepsy after anterior temporal lobectomy (Ji et al., 2015), and in individuals with PD after deep brain stimulation (van Hartevelt et al., 2014).

### Cognitive-Network Correlations

Cognitive-network analyses help to validate group-level structural differences. We examined key cognitive domains compromised in PD: processing speed, working memory, and episodic memory. Our observations suggest that PD-MI had more correlations between neuropsychological composites and connectivity indices than PD-Well and non-PD peers. For PD-Well, correlations were found between working memory and node strength, as well as for working memory and path length. The PD-Well group’s working memory showed a negative correlation with node strength and path length (i.e., higher node strength and path lengths were associated with lower working-memory scores). The topological changes suggest a possible maladaptation in the brain networks of those with PD, as suggested in other neurological disorders (Doucet et al., 2015; Drakesmith et al., 2015; Wu et al., 2009). Thus, these topological indices might be used as markers for cognitive performance in PD.

There were no significant local network correlations between the network indices and neuropsychology composites in the control group (Figure 5). This negative finding is expected given this group’s relative cohesiveness, intact performance, and restricted range of scores. In contrast, the PD-Well group showed an increased number of strong correlations, while the PD-MI showed an even higher number of strong correlations. The increase in the number of correlations might reflect a progressive alteration of brain connectivity mediating the cognitive decline. Although we observed increasing numbers of correlations between network indices and neuropsychological composites, we did not find verbal memory correlations to any of the network indices. The lack of correlations between verbal memory and network indices in PD-MI was possibly due to the restricted range of their scores (all impaired).

### Study Strengths and Limitations

Overall, the study strengths include the presentation of a comprehensive correlational analysis between network topology and cognition using various neuropsychological indices, prospective PD and control matching on demographic variables (i.e., age and education), and robust identification of memory impairment in PD. The PD subgroup cognitive profiles showed expected reductions in processing speed for PD relative to non-PD controls, with PD-MI showing reductions in memory and processing speed. In this work, we used a weighted network method that yields more stable topological metric results than binary network methods and is robust despite graph density differences; hence, it does not require thresholding to generate the connectomes (Colon-Perez et al., 2016). Topological features of binary network connectomes may be affected by their graph density and ultimately may hinder comparisons between groups (Langer, Pedroni, & Jancke, 2013; Sporns, 2011b). We could circumvent the problem of thresholding by employing a weighted framework that reduces the effects of thresholding in network indices results (Colon-Perez et al., 2016).

The authors recognize some of the study limitations. MR tractography is susceptible to false positives and false negatives; hence, care is required when analyzing and interpreting tractography-derived results (Alhourani & Richardson, 2015). In this study, we used a different edge weight to calculate the strength of connectivity between nodes. This approach reduces the tractography bias effects of seed density and length (Colon-Perez, Spindler, et al., 2015). Also, the edge weight is defined only along the path of the streamlines, which reduces the possibility of obtaining streamlines from extraneous areas. Also, the use of a weighted network defines connectomes as a set of weak and strong connections, which has been shown to reduce the effects of false-positive connections in the quantification of connectome indices using binary networks (Colon-Perez et al., 2016). Moreover, false-positive connections yield connectome edges with a small number of streamlines, which in turn corresponds to a low edge weight value; these spurious links will only produce changes in node strength of less than 1% (Colon-Perez et al., 2016). Another limitation is the relatively low spatial resolution (2 mm isotropic for diffusion images) in tractography, which is a limiting factor when estimating small tracts and may lead to false negatives (Colon-Perez, King, et al., 2015; Ford et al., 2013). In the current work, we used high angular-resolution diffusion imaging to increase the angular resolution and enable better estimation of the fiber orientation on each voxel (Jian et al., 2007; Tuch et al., 2002). It also has been reported that the connectome changes with age (Wu et al., 2007). However, the mean and range of age in each group is similar to ensure there were no age differences between groups. Thus, the current results may only apply to similarly aged populations. We also did not consider genetics, which has been shown to modulate connectome topology in patients with PD through the rs405509 risk allele (TT) (Shu et al., 2015). An additional limitation is the relatively small sample size of this study, particularly within the PD-MI group (*n* = 9). Future studies are encouraged using larger sample sizes and additional explorations of network differences in memory versus other cognitive (e.g., executive working, attention) deficits.

## CONCLUDING REMARKS

Our connectome analyses suggest a loss and reorganization of brain white matter structure in PD, particularly PD with memory impairment. We identified a reduction of connectome topological and neuropsychological indices. Our results show a relationship between cognitive deficits and connectome structure alteration in PD with mild cognitive impairments. It remains to be determined whether the observed network changes are causal of the neurocognitive deficits or vice versa. Also, further studies are needed to assess the mechanisms relating to the observed topological changes in brain structure to the neurocognitive deficits. The data suggest a broader change, at the level of the connectome, associated with the clinical manifestations of cognitive phenotypes, particularly the memory phenotype of PD.

## ACKNOWLEDGMENTS

We are grateful to the participants involved in the current investigation. We are also grateful to Tony Mancuso, MD, for his help in securing the MR Siemens Verio, and the UF Radiology team for their guidance. We would like to acknowledge Irene Malaty, MD, Ramon Rodriguez, MD, Janet Romrell, ARNP, Pam Zeilman, ARNP, and all the faculty in the UF Center for Movement Disorders and Neurorestoration, Gainesville, Florida, for referring individuals to the investigation. We also thank Jessica Williams, Cassie Catania, Jade Ward, Katie Rodriguez, and Breana Wallace, for their assistance with participant recruitment.

## AUTHOR CONTRIBUTIONS

Luis M. Colon-Perez: Conceptualization; Formal analysis; Investigation; Methodology; Project administration; Software; Writing – original draft; Writing – review & editing. Jared J. Tanner: Conceptualization; Data curation; Formal analysis; Methodology; Writing – original draft; Writing – review & editing. Michelle Couret: Formal analysis; Investigation; Writing – review & editing. Shelby Goicochea: Formal analysis; Investigation. Thomas H. Mareci: Project administration; Supervision; Conceptualization; Methodology; Writing – review & editing. Catherine C. Price: Conceptualization; Formal analysis; Funding acquisition; Investigation; Methodology; Project administration; Supervision; Writing – original draft; Writing – review & editing.

## FUNDING INFORMATION

Funding for this research was supported by the National Institutes of Health (grant nos. K23 NS060660, RO1 NR014181, and R01 NS082386), by the National Institutes of Health Clinical and Translational Science Award program (grant nos. UL1TR001427, KL2TR001429, and TL1TR001428), and by the UF Center for Movement Disorders and Neurorestoration and the Brain and Spinal Cord Injury Research Trust Fund of the State of Florida. The content is solely the responsibility of the authors and does not necessarily represent the official views of the National Institutes of Health.

## TECHNICAL TERMS

Structural connectome: Brain connectivity graphs obtained from diffusion MRI and tractography.
Tractography: Three-dimensional representation of the brain’s white matter tracts derived from diffusion MRI data.
Dementia Rating Scale-2: A screening measure used to assess a patient’s overall level of cognitive functioning.
Unified Parkinson’s Disease Rating Scale: The most commonly used measure of Parkinson’s disease clinical symptom severity.
Prodromal dementia: The earliest stage of neurodegenerative disease when there is a decline in memory or cognition, but functional independence remains intact.
Edge weight: Graph theoretical representation of the strength of the pairwise connections between the nodes in a graph.
Characteristic function: In probability and statistics, a function that precisely defines the members of a set. It takes a value of one for a member of the set and zero for a nonmember.
Cognitive composites: Averages of standardized scores from multiple neurocognitive measures theoretically measuring the same cognitive domain.
Global network indices: Average value of a network index across all nodes for a single subject.
Local network indices: Value of a network index for every node and a single subject.
