# Supplementary material for: Cognition and connectomes in nondementia idiopathic Parkinson’s disease
Source: Netw Neurosci. 2018 Mar 1;2(1):106–24. doi: 10.1162/NETN_a_00027 (PMC5989988; doi:10.1162/NETN_a_00027)
Supplement: Supplemental Figures [file netn-02-106-s001.pdf]

Colon-Perez, L.M., Tanner, J. J., Couret, M., Goicochea, S., Mareci, T. H., & Price, C. C. (2017). Supplement for "Cognition and connectome in non-dementia idiopathic Parkinson's disease." *Network Neuroscience*. 2*(1)*: 106−124.
https://doi.org/10.1162/netn_a_00027

Supplemental Figures

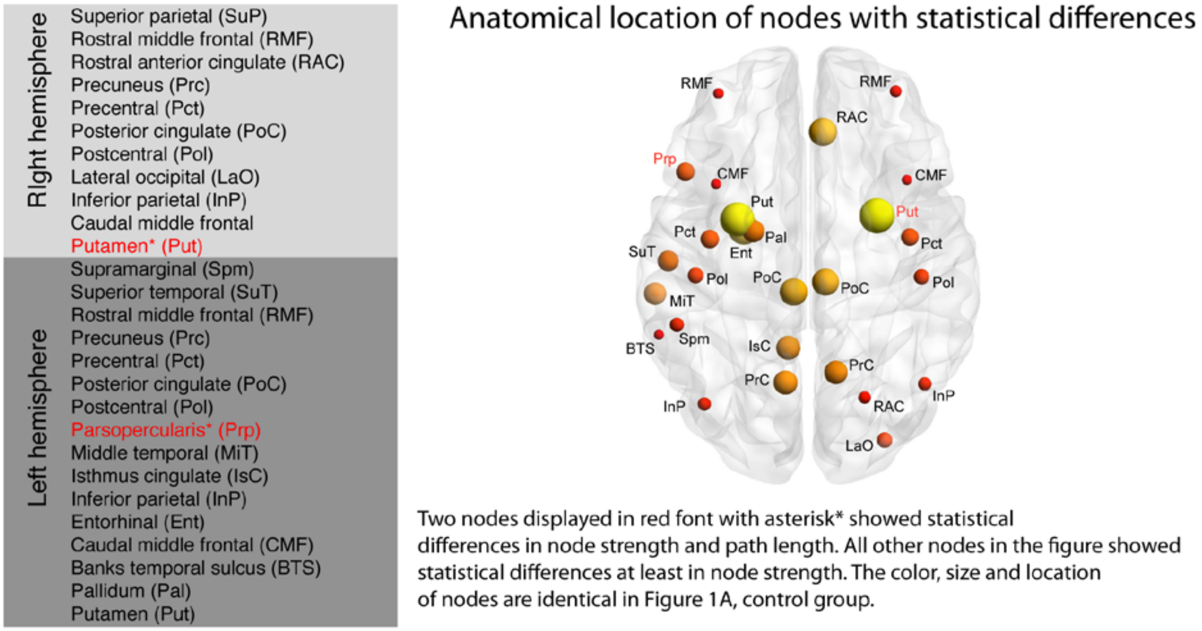

Supplemental Figure 1. Anatomical location of nodes with reduced network connectivity indices on Parkinson's disease participants with mild cognitive impairment (See Figures 3 and 4). *Note: Banks temporal sulcus is an abbreviation for the banks of the superior temporal sulcus region, which is the name in the free surfer nomenclature and the full name is used in the main text.

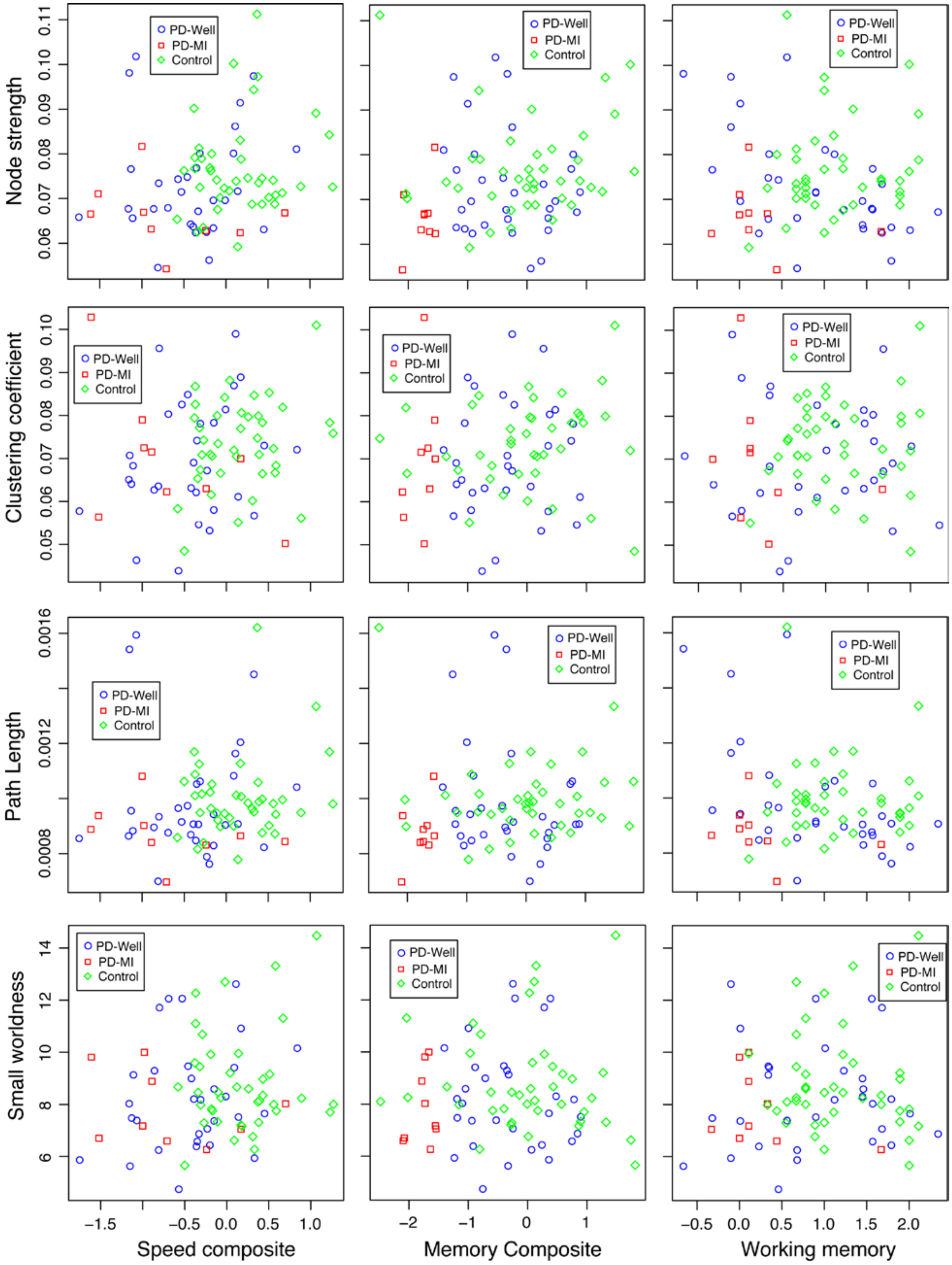

Supplemental Figure 2. Scatter plots of correlation data displayed in Table 2.

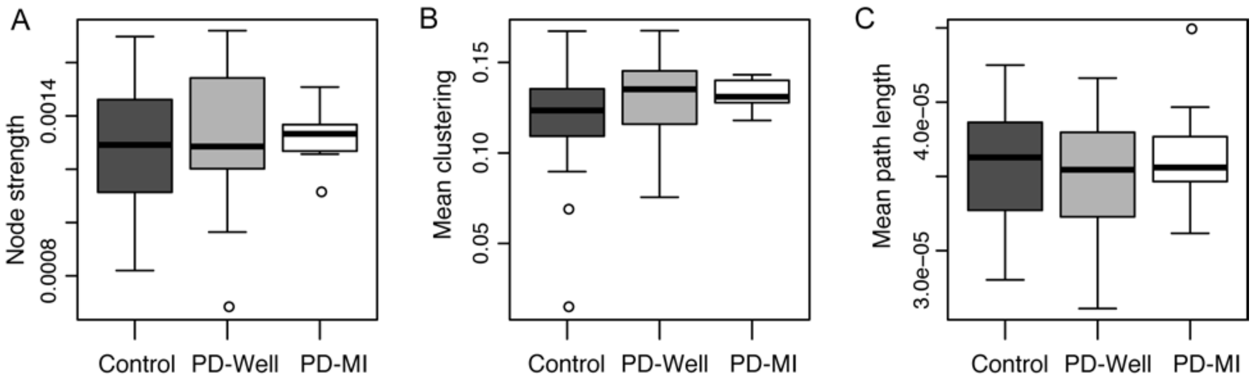

Supplemental Figure 3. Boxplots of global network indices using FA as the edge weight. (A) Average node strength per brain averaged across subjects within each group. (B) Mean clustering = mean global clustering coefficient per brain averaged across subjects within each group. (C) Mean path length = mean global path length per brain averaged across subjects within each group. (^{o} = outliers). Statistics performed with a nonparametric Mann-Whitney test and no differences were found between any group.

ONLINE DATABASES

As an online supplement, we provide the data of this study organized as an R database. Interested readers can download R from this link (https://cran.r-project.org/). The naming nomenclature is kept the same on both databases. The results shared on these databases are the global (one values per brain) and the local (one value per node per brain). Below is the legend of variables in the R databases.

con = control, pd = PD-Well, pd_am = PD-MI, str = node strength, ccw = clustering coefficient, plw = path length, db = graph density, global or glob = global results for all participant in an specific cohort (i.e., con, pd or pd_am), all = array containing global values of all cohorts (first row = control, second = PD-Well, third = PD-MI), contDens = results controlling for global graph density, p = p value, MW = Mann Whitney, NeuPsy = neuropsychological composites, nuisance_Var = nuisance variables (e.g., head motion, slice dropuout, TICV = total intracranial volume, etc), vol = volume of brain regions, SA = surface area, swW = small worldness, ave = average across the members of a cohort.

Command for the Mann Whitney test in R

```
wilcox.test(<cohort 1>, <cohort 2>,exact=FALSE)$p.value
```

Load the fdr tool library (https://cran.r-project.org/web/packages/fdrtool/fdrtool.pdf)

```
library(fdrtool)
```

Command to correct the p values using fdrtool

```
fdrtool(<uncorrected p list>,statistic="pvalue",cutoff.method="fndr")
```
